# Retraction Note to: microRNA-128 mediates CB1 expression and regulates NF-KB/p-JNK axis to influence the occurrence of diabetic bladder disease

**DOI:** 10.1186/s12967-021-02719-3

**Published:** 2021-02-08

**Authors:** Xin Gou, Jing Wu, Mingqing Huang, Yuting Weng, Tongxin Yang, Tao Chen, Guiqing Li, Kewei Fang

**Affiliations:** 1 Department of Urology, The Second Affiliated Hospital of Kunming Medical University, No. 374, Dianmian Dadao, Kunming, 650101 Yunnan People’s Republic of China; 2 Department of Biochemistry and Molecular Biology, The Primary Medicine School of Kunming Medical University, 650101 Kunming, People’s Republic of China; 3 Department of Urology, The 2Nd Hospital of Kunming Medical University, Kunming, 650101 People’s Republic of China

## Retraction Note: J Transl Med (2020) 18:284 https://doi.org/10.1186/s12967-020-02406-9

This article [1] has been retracted by the authors. The corresponding author has stated that part of the research, including western blot and qPCR experiments, had been outsourced to a third party company that has gone out of business, and the authors have been unable to reproduce the results themselves. Therefore the authors no longer have confidence in the validity of the data and the conclusions drawn. In addition, contrary to the statement in the article, the animal experiments have not been approved by the Animal Ethics Committee of the Second Affiliated Hospital of Kunming Medical University. All authors agree with this retraction.
